# Somatic Embryogenesis and Plant Regeneration from Commercial Soybean Cultivars

**DOI:** 10.3390/plants9010038

**Published:** 2019-12-25

**Authors:** Ghulam Raza, Mohan B. Singh, Prem L. Bhalla

**Affiliations:** Plant Molecular Biology and Biotechnology Laboratory, Faculty of Veterinary and Agricultural Sciences, The University of Melbourne, Parkville, VIC 3010, Australia; graza4@gmail.com (G.R.); mohan@unimelb.edu.au (M.B.S.)

**Keywords:** legume, soybean cv. Snowy, tissue culture

## Abstract

The efficient regeneration of plants from commercial genotypes is a pre-requisite for successful genetic transformation, to apply modern crop improvement techniques such as CRISPR-based genome editing. Plant regeneration through the somatic embryogenesis pathway offers an advantage over the organogenesis approach, avoiding the risk of developing chimeras. Plant genotype, explant type, and media compositions play an essential role in the in-vitro regeneration of plants. This study aimed to characterize the commercially grown Australian soybean genotypes for their potential to induce somatic embryos, embryo proliferation, maturation, germination, and plant regeneration. Overall, nine soybean cultivars belonging to different maturity groups were evaluated. Immature cotyledon ranging from 2–4 and 4–6 mm in size were used as explants for somatic embryogenesis induction. Maximum somatic embryo induction frequency (86%) was observed from 4–6 mm immature cotyledons of the cv. Jack (MG III), followed by 66%, 26%, 21%, and 6% in cultivars Williams (MG III), Snowy (MG III), MoonB1 (MG V), and PNR791 (MG V), respectively. On the other hand, cv. Snowy showed maximum somatic-embryo-inducing potential (67%) in 2–4 mm immature cotyledons followed by Williams, Jack, MoonB1, and PNR791. Somatic embryos from Jack, Williams, and Snowy cultivars were further tested for embryo proliferation, maturation, and germination. Maximum proliferation and maturation were observed in cv. Jack, followed by Snowy and Williams. However, cv. Snowy showed a significantly higher conversion of cotyledonary stage embryos to plantlets (85%), than both Jack and Williams cultivars (53% each). In conclusion, this study outlined a protocol for somatic embryogenesis and plant regeneration from three soybean cultivars. Our findings suggest commercial cv. Snowy could be a good candidate for developing transgenic plants through somatic embryogenesis.

## 1. Introduction

Soybean (*Glycine max* L.) is an important agricultural crop. It ranks 7th in terms of revenue, with an income of ~121 billion US dollars in the year 2016 [1]. Soybean seeds are rich in protein, oil, and unsaturated fatty acids. Further, soybean is also a good source of minerals, B vitamins, and isoflavones. Soybean is also extensively used in animal feed as well as in other industrial applications [2,3,4]. Fertile soybean plants have been regenerated in vitro, mainly through organogenesis [5,6]. There is a high risk of obtaining chimeric plants through organogenesis, during genetic transformation. On the other hand, plant regenerated via somatic embryogenesis arises from a single cell, avoiding the complexities of chimeras. Therefore, the development of transgenic soybean plants through somatic embryogenesis has been proposed as a reliable method for recovering stable transgenic lines [7,8]. A number of studies on in vitro regeneration collectively indicated that the initiation of the somatic embryos from explants and plant regeneration depends on the genotypes or cultivar [9,10,11,12,13,14,15]. Similar to other important crop species, the efficiency of plant regeneration in soybean is highly genotype-dependent, and efficient regeneration has only been reported from Jack, a cultivar of inferior agronomical value [11,16,17,18]. A number of studies have explored the regeneration potential of different commercially grown soybean cultivars [6,13,14,19,20,21,22], and the embryogenic genotypes found belonged to MG 00-III maturity groups. These cultivars are primarily adapted to the climatic conditions of Canada, the USA, and Brazil [9,19,23,24,25].

In Australia, soybean was adopted as a commercial crop in the early 1950s. Currently, soybean is commercially grown in Queensland, New South Wales (NSW), Northern Victoria, and Western Australia. Cultivars such as Snowy, Bunya, A6785, Fernside, Warrigul, Mooni, and Jabiru are favored for cultivation. Snowy is the first cultivar adapted in the Riverina region of NSW because of its good yield and seed quality. The Snowy crop matures early, a trait that helps to avoid frosts in Victoria. Moreover, Snowy, with a high protein content of above 40%, is used for tofu and soymilk production, providing farmers about $200/ton premium compared to other soybean cultivars that are used for oil extraction. Bunya is a quick maturity cultivar, while A6785 belongs to the medium maturity group. Both Bunya and A6785 are grown in Queensland and are resistant to the two main races of Phytophthora root rot found in this state. Bunya is suitable for the manufacturing of tofu, and the A6785 variety is chosen for soy flour and soymilk. A6785 is also recommended in an area where crops are susceptible to weather damage around harvesting time. Although the A6785 cultivar has marginally lower protein amounts, its cultivation at the right time results in high seed yields. Bunya produces large-sized seeds that are susceptible to damage during harvesting. Fernside, with an excellent grain size, is a medium maturing variety and has replaced the A6785 cultivar in the edible market. Moonbi cultivar is a fast-maturing type and is recommended for cultivation in NSW regions. It has high a protein content and better weather tolerance [6,26,27]. 

Photoperiod and temperature play a critical role in determining the soybean phenology, adaptation, and yield [28,29]. Hence, soybean varieties are adapted in restricted regions of Australia, limiting the availability of the suitable soybean cultivars in potential growing regions. Therefore, there is a need to breed soybean cultivars with a wide range of adaptability with improved quality and agronomic traits.

We aim to extend the genetic transformation technology for commercial soybean varieties for the application of the CRISPR-based genome editing technology. Identification of the most suitable commercial variety, which might be regenerated through somatic embryogenesis, is the first step, as plant regeneration is highly genotype-dependent and to-date only a model cv. Jack has been shown to amenable to in vitro regeneration. In the present study, we found that commercial cultivar Snowy has a better regeneration potential through somatic embryogenesis than the Jack model variety. This study provides the first report on regeneration of an Australian commercial cultivar via somatic embryogenesis. Hence, the Snowy cultivar could be used for functional genomics studies using the newly developed CRISPR technology.

## 2. Results and Discussion

**Figure 1:**
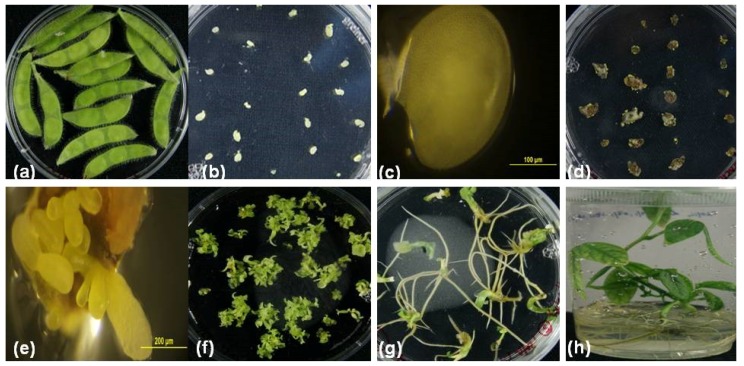
Steps in regenerating soybean plants through somatic embryogenesis (cv. Snowy is shown as an example), (**a**) immature pods for isolation of immature cotyledons; (**b**) immature cotyledons cultured on the D40 medium; (**c**) close view of the isolated immature cotyledons; (**d**) induction of somatic embryos after two months of culturing, (**e**) close view of somatic embryos, (**f**) proliferated histo-differentiated mature embryos, (**g**) germinated embryos, and (**h**) the rooted plants.

In the present study, we identified the plant regeneration response of nine soybean cultivars, including six commercially grown Australian cultivars through somatic embryogenesis. Figure 1 depicts the steps for regenerating soybean plants through somatic embryogenesis. Successful somatic embryos induction and their regeneration into plants were obtained in three soybean genotypes. The highest regeneration efficiency was obtained in cv. Snowy, compared to the model cvs. Jack and Williams (Table 1). Table 1 summarizes the cultivars with the respective maturity groups, the total number of explants (sum of two explant sources) used from each cultivar, and the number of regenerated plants obtained during this study. Our literature search showed that our study was the first report on the embryogenic and regenerative potential of Australian commercial cultivar, Snowy. The Development of transgenic plants through somatic embryogenesis lays the foundations for CRISPR studies, as the genes of interest can be manipulated directly in a commercial variety. Moreover, Snowy is an important cultivar in Australia, especially for growers in the Riverina region. Snowy seeds are valued for making tofu and soymilk. Due to these features, seeds of Snowy attract a premium price as compared to other soybean cultivars sold for oil.

Cultivar genetic, maturity group, and explant (immature cotyledon) size played an essential role in the regeneration of soybean plants via somatic embryogenesis. In this study, fully developed soybean plants were obtained only from early-maturing soybean cultivars Snowy (MG III), Jack (MG II), and Williams (MG III), while no regeneration was achieved in late-maturing cultivars Bunya (MG V), PNR791 (MG V), A6785 (MG VI), MoonB1(VI), Bragg (MG VII), and Fernside (MG VII). Earlier, the maturity group effect on somatic embryogenesis of soybean was reported in several studies. Bailey et al. [25] reported that there was no correlation between maturity and somatic embryogenesis. On the contrary, other investigators [20,30,31] showed that soybean cultivars in early maturity groups (MG 00 to MG 1) produced more somatic embryos than later cultivars of later maturity groups. In the present study, cultivars MG II and MG III displayed significantly better somatic embryogenesis than cultivars of later MG IV-VII. This study was in agreement with Ko et al. [21], who reported higher embryogenic responses in genotypes of maturity groups MGII-MGIV, under hygromycin selection than cultivars of early and later maturity groups (MG 00-MG I) and (MG V-MG VIII). 

**Figure 2:**
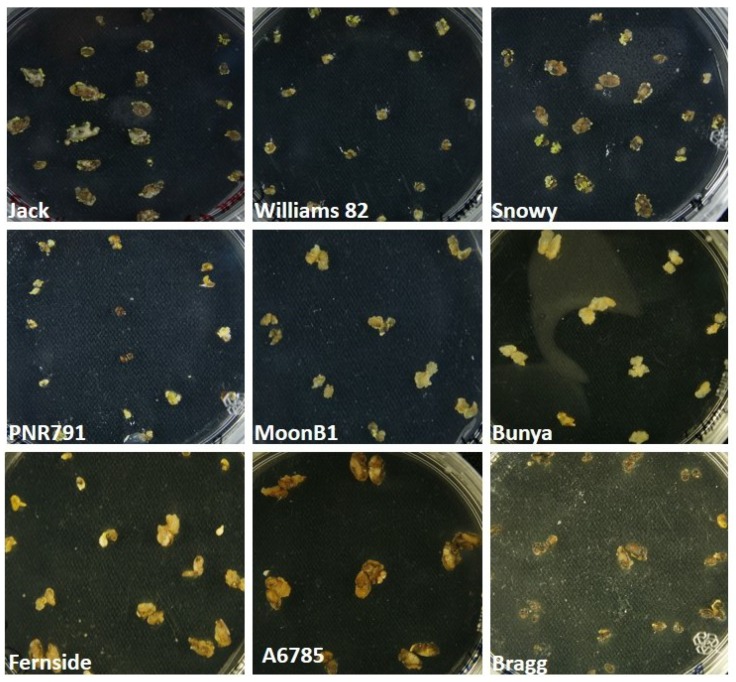
Explants from different soybean cultivars during somatic embryos induction.

During our study, the flowering initiation time varied in different cultivars. The cultivars Jack, Williams, and Snowy initiated the flowering almost at the same time because these cultivars grouped in lower maturity groups. Therefore, the explants collection time was the same for these cultivars while it was varied for the other cultivars. The isolated immature cotyledons also varied in size. Thus, the immature cotyledons were classified into two groups according to size (Group 1: 2–4 mm, Group 2: above 4–6 mm). To induce the somatic embryos, immature cotyledons were cultured on a medium containing a high concentration of 2, 4-D (40 mg L^−1^). Initially, all explants looked dead and of different colors, such as light brown and black. The damage looked worse on large immature cotyledons. Such a condition was observed in all cultivars. Despite this, the explants were sub-cultured on a fresh D40 medium. Within 30 days of culturing, somatic-embryo induction was observed from the black and brown explants. The cultivar Jack was the first in the initiation of somatic embryos from the abaxial side, followed by Snowy and Williams, while the other cultivars showed a very low induction. Cv. Jack initiated somatic embryos at the margins of explants, while in Cv. Snowy, the whole upper surface produced a bunch of globular embryos (Figure 2). Jack produced more single globular structures while Snowy and Williams developed somatic embryos in a cluster. The induced embryos were bright green, yellow, and light brown. The structure and location of somatic embryos on explants were in agreement with Ko et al. [21], who characterized somatic embryos as green, globular, and translucent.

Somatic embryogenesis responses of all cultivars were significantly different even within the cultivars of the same maturity group. Earlier reports have highlighted the differences among soybean cultivars in somatic embryogenesis from immature cotyledons [7,12,13,19,20,25,32,33,34].

**Figure 3:**
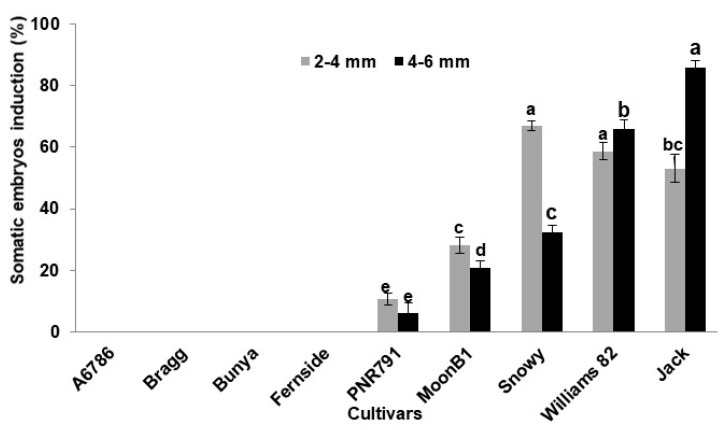
Comparison of somatic embryos induction of soybean cultivars from two different sizes of immature cotyledon explants. Data points represent the mean ± SE of three replicates. Column values with different letters are statistically significant at *p* < 0.05.

In this study, maximum somatic embryogenesis response (86%) was observed from 4–6 mm immature cotyledons of cultivar Jack (MG III), followed by 66%, 26%, 21%, and 6% from cultivars Williams (MG III), Snowy (MG III), MoonB1, and PNR791, respectively (Figure 3). On the other hand, the cultivar Snowy (MG III) showed a maximum somatic-embryo-inducing potential (59%) in 2–4 mm immature cotyledons followed by Jack, Snowy, MoonB1, and PNR791 (Figure 3). However, cultivars A6785, Bragg, Bunya, and Fernside did not induce somatic embryos from both types of immature cotyledon explants (Figure 2 and Figure 3). These findings are in line with Yang et al. [12], who reported a significantly varied response for somatic embryogenesis from explants of different sizes (<3, 4–5, 6–8, and >8 mm).

Earlier, it was reported that the development stage and the physiological condition of explants play a vital role in somatic embryogenesis [35], which is why different explants of different sizes show different somatic embryo induction response. Differences in somatic embryos production among cultivars could also be due to the varying concentrations of ABA in explants of different cultivars, as it was observed earlier that the larger immature cotyledons exhibited higher ABA levels compared to the smaller ones [12].

**Figure 4:**
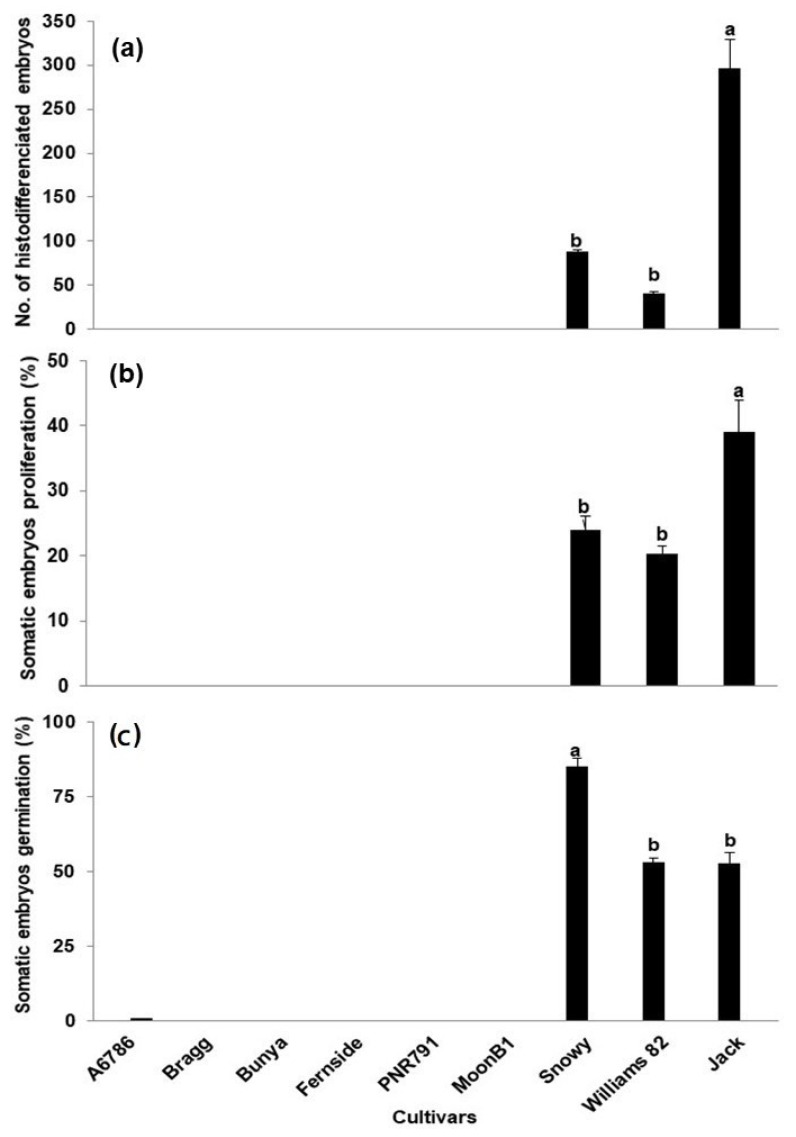
Proliferation, maturation, and germination of somatic embryos in soybean cultivars, (**a**) number of proliferated somatic embryos, (**b**) number of matured somatic embryos, and (**c**) germination percentage of matured somatic embryos. Data points represent the mean ± S.E. of three replicates. Column values with different letters are statistically significant at *p* < 0.05.

**Figure 5:**
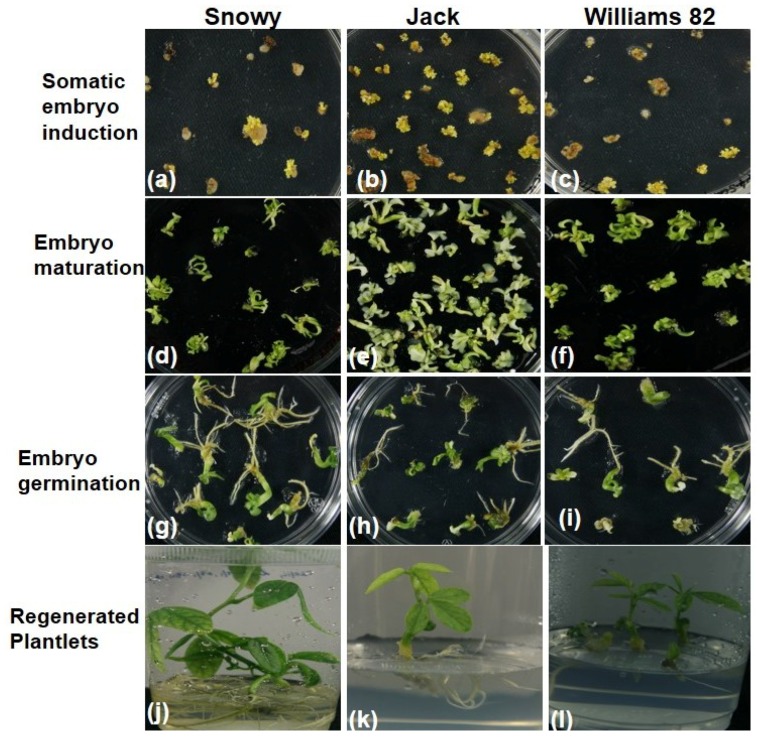
Comparison of cv. Snowy with Jack and Williams 82 for somatic embryogenesis, (**a**–**c**) induction, (**d**–**f**) maturation, (**g**–**i**) germination, and (**j**–**l**) regenerated soybean plants.

The induced embryos were further cultured on the D20 medium to observe the proliferation of somatic embryos. Regardless of extensive attempts, the proliferative cultures of six cultivars could not be established. Only three cultivars, Jack, Williams, and Snowy, showed the somatic embryo proliferation (Figure 4). Cultivar Jack proliferated the most, followed by Snowy and Williams, respectively (Figure 4a). These observations also reflect the variability in the soybean embryo proliferation phase. For histo-differentiation from globular to the cotyledonary stage, the proliferated somatic embryos were cultured on a hormone-free MS medium containing 0.5% activated charcoal. The proliferation response on this medium was better, compared to the D20 medium. The maximum number of histo-differentiated embryos was obtained from the cultivar Jack (297), followed by Snowy (88) and Williams (41) (Figure 4b). When histo-differentiated embryos were recorded, different abnormal embryo phenotypes, such as long hypocotyl, cup-shaped cotyledons, and fused cotyledons were also observed in all cultivars. These phenotypes were more in cvs. Jack and Williams than Snowy. Due to this, Snowy showed the highest cotyledonary stage somatic embryos germination efficiency (85%) compared to 52% for both Jack and Williams (Figure 4c). The comparison of all stages involved in the regeneration of soybean plants through somatic embryogenesis is depicted in Figure 5.

On the whole, the maximum number of regenerated soybean plants were obtained from Snowy, followed by Jack and Williams (Table 1). It is worth mentioning that the variations at different phases of regeneration, such as embryo induction and germination, were also observed among the cultivars of the same maturity group. Furthermore, this study also indicated the absence of correlation between a number of histo-differentiated embryos and frequencies of conversion from the cotyledonary stage embryos to plantlets, in the examined genotypes. This observation was not in agreement with Bailet et al. [36], who reported that cultivars with a greater production of well-differentiated embryos showed a high conversion efficiency from the cotyledonary stage of somatic embryos to plantlets. The conversion efficiency might be further improved by replacing the differentiation medium MSM6AC with a liquid medium, which has been modified for differentiation and maturation of soybean somatic embryos [18].

In conclusion, we report that Snowy is the most efficient genotype for plant regeneration through somatic embryogenesis among the tested commercially grown Australian cultivars. This cultivar induces more somatic embryos from smaller size immature cotyledons (2–4 mm) than larger immature cotyledons. Although the efficiency of somatic embryo induction and proliferation of this cultivar was less than the reference cultivar Jack, its conversion efficiency from the cotyledonary stage somatic embryos to plantlets could make this cultivar useful for genetic transformation. Its intermediate level of somatic embryos induction might be improved by changing the culture conditions, such as the auxin concentration in the medium. We are now testing this cultivar for efficient plant transformation and regeneration.

## 3. Materials and Methods

### 3.1. Plant Material

Seven commercial (Bunya, Fernside, Snowy, A6786, PNR791, Bragg) and two model cultivars (Jack, Williams) of soybean (Glycine max L. Merr.) were used for somatic embryogenesis and plant regeneration. Seeds of Bunya, Fernside, Snowy, A6786, and PNR791 cultivars were obtained from Maralong (Pvt.) Ltd. PB-Agrifood, Brisbane, Australia. The seeds were sown in pots containing a seed raising mix. The pots were placed in a controlled growth cabinet at 25 ± 2 °C with a 14 h photoperiod and light intensity of 600–1500 moles/m^2^/s. The photoperiod was reduced to 10 h after one month, to induce flowering. After 2–3 weeks of flower induction, healthy pods from each cultivar were collected and sterilized by washing with sterile ddH_2_O containing few drops of Tween-20 for five minutes, followed by disinfection with 10% commercial bleach solution (8%–12 % chlorine) for 20 min, and was rinsed with sterile ddH_2_O five times, to remove the excess sodium hypochlorite [14]. The immature cotyledons were retrieved and the embryo axes were removed, following the method described by Lazzari et al. [37]. The sizes of cotyledon explants were categorized into two groups: 2–4 mm and 4–6 mm. The explants were placed with the abaxial side facing down on the D40 medium containing MS salts [38], B5 vitamins [39], 100 mg L^−1^ myo-inositol (I3011; Sigma-Aldrich), 30 g L^−1^ sucrose, 40 mg L^−1^ 2,4-D (D8407; Sigma-Aldrich), 2 g L^−1^ Gelrite (G1910; Sigma-Aldrich), and pH 7.0. The plates were incubated at 25–27 °C, with 25–40 µmol m^−2^ S^−1^ light on a 16/8 h light/dark cycle. After 45 days in a D40 medium, somatic embryo induction from each explant was noted, and each cotyledon was visually scored [5,10]. The induced somatic embryos were dissected and further cultured on the D20 medium comprising MS salts, B5 vitamins, 100 mg L^−1^ myo-inositol, 30 g L^−1^ sucrose, 660.6 mg L^−1^ asparagine (A4159; Sigma-Aldrich), 20 mg L^−1^ 2,4-D, 2 g L^−1^ Gelrite, and pH 5.7. Sub-culturing on a fresh D20 medium occurred every two weeks. After six weeks, somatic embryo clumps were counted and further cultured on the M6AC medium, as described by Finer [5]. After 30 days, matured embryos were counted and sub-cultured on the M6AC medium again, until the embryos turned to a cream color. Then, the embryos were desiccated for three days by placing the 15 embryos in an empty Petri plate wrapped with parafilm. The desiccated embryos were further cultured on an embryos germination medium [MS salts, B5 vitamins, 100 mg L^−1^ myo-inositol, 30 g L^−1^ sucrose, 2 g L^−1^ Gelrite, and pH 5.8]. After six weeks, the percentage of germinated embryos converted into complete plants was calculated. The rooted plants were cultured in sterile plastic jars for further growth. Fully regenerated soybean plants were transferred to pots containing commercially available potting mix, under glasshouse conditions. 

### 3.2. Statistical Analysis

The experiments were done using a completely randomized design with three replicates. All data of somatic embryos induction, proliferation, and germination were analyzed by one-way analysis of variance followed by a Fisher’s protected LSD test and was considered significant at *p* < 0.05. The analysis was carried out using the statistical software Genstat, 15th Edition [40]. 

## Figures and Tables

**Table 1:** Summary of somatic embryogenesis response and regenerated soybean plants. Values with different lowercase letters are significantly different at *p* < 0.05.

Soybean Cultivars	Maturity Group	Total no. of Explants (Both Sizes)	Somatic Embryos Induction (%) (Mean ± S.E)	Proliferated Embryos (Mean ± S.E)	Embryos on Germination Medium	Gemination (%) (Mean ± S.E)	Plants Obtained
Snowy	III	145	36 ± 2.59 ^c^	88 ± 2.31 ^b^	50	85 ± 2.64 ^a^	42
Jack	II	141	69 ± 2.38 ^a^	297 ± 32.05 ^a^	50	53 ± 3.54 ^b^	27
Williams	III	131	62 ± 2.91 ^b^	41 ± 2.08 ^c^	50	53 ± 1.61 ^b^	26
MoonB1	V	130	24 ± 2.38 ^d^	0	0	0	0
PNR791	V	120	8 ± 2.60 ^e^	0	0	0	0
A6785	VI	154	0	0	0	0	0
Bunya	VI	168	0	0	0	0	0
Bragg	VII	70	0	0	0	0	0
Fernside	VII	134	0	0	0	0	0

## References

[1] FAO (2016). Statistical Yearbook.

[2] Birt D., Hendrich S., Anthony M., Alekel D., Boerma H.R., Specht J.E. (2004). Soybeans and the prevention of chronic human disease. Soybeans: Improvement, Production and Uses.

[3] Wilson R., Boerma H.R., Specht J.E. (2004). Seed composition. Soybean: Improvement, Production and Uses.

[4] Raza G., Ahmad N., Hussain M., Zafar Y., Rahman M. (2016). Role of Genetics and Genomics in Mitigating Abiotic Stresses in Soybeans. Environmental Stresses in Soybean Production.

[5] Finer J.J. (2016). Generation of transgenic soybean (Glycine max) via particle bombardment of embryogenic cultures. Curr. Protoc. Plant Biol..

[6] Raza G., Singh M.B., Bhalla P.L. (2017). In vitro plant regeneration from commercial cultivars of soybean. Biomed. Res. Int..

[7] Droste A., Pasquali G., Bodanese-Zanettini M. (2002). Transgenic fertile plants of soybean [*Glycine max* (L.) Merrill] obtained from bombarded embryogenic tissue. Euphytica.

[8] Homrich M.S., Passaglia L.M.P., Pereira J.F., Bertagnolli P.F., Pasquali G., Zaidi M.A., Altosaar I., Bodanese-Zanettini M.H. (2008). Resistance to Anticarsia gemmatalis Hübner (Lepidoptera, Noctuidae) in transgenic soybean (*Glycine max* (L.) Merrill Fabales, Fabaceae) cultivar IAS5 expressing a modified Cry1Ac endotoxin. Genet. Mol. Biol..

[9] Santarem E., Pelissier B., Finer J. (1997). Effect of explant orientation, pH, solidifying agent and wounding on initiation of soybean somatic embryos. In Vitro Cell. Dev. Biol. Plant.

[10] Meurer C.A., Dinkins R.D., Redmond C.T., McAllister K.P., Tucker D.T., Walker D.R., Parrot W.A., Trick H.N., Essig J.S., Frantz H.M. (2001). Embryogenic Response of multiple soybean [*Glycine max*(L.) Merr.] cultivars across three locations. In Vitro Cell. Dev. Biol. Plant.

[11] Walker D., Parrott W. (2001). Effect of polyethylene glycol and sugar alcohols on soybean somatic embryo germination and conversion. Plant Cell Tissue Organ Cult..

[12] Yang C., Zhao T., Yu D., Gai J. (2009). Somatic embryogenesis and plant regeneration in Chinese soybean (*Glycine max* (L.) Merr.)—Impacts of mannitol, abscisic acid, and explant age. In Vitro Cell. Dev. Biol. Plant.

[13] Droste A., Silva A.M.D., Souza I.F.D., Strohm B.W., Neto L.B., Bencke M., Sauner M.V., Zanettini M.H.B. (2010). Screening of Brazilian soybean genotypes with high potential for somatic embryogenesis and plant regeneration. Pesqui. Agropecu. Bras..

[14] Texeira L.R., Braccini A.D.L.E., Churata B.G.M., Vieira E.S.N., Martins P.K., Schuster I. (2011). Evaluation of soybean cultivars on the embryogenic and organogenic potential. Acta Sci. Agron..

[15] Thankaraj Salammal M., Vasudevan Ramesh A., Shu-Ye J., Andy G., Srinivasan R. (2013). In vitro Regeneration and Genetic Transformation of Soybean: Current Status and Future Prospects. A Comprehensive Survey of International Soybean Research—Genetics, Physiology, Agronomy and Nitrogen Relationships.

[16] Samoylov V.M., Tucker D.M., Parrott W.A. (1998). Soybean [*Glycine max* (L.) merrill] embryogenic cultures: The role of sucrose and total nitrogen content on proliferation. In Vitro Cell. Dev. Biol. Plant.

[17] Samoylov V.M., Tucker D.M., Thibaud-Nissen F., Parrott W.A. (1998). A liquid-medium-based protocol for rapid regeneration from embryogenic soybean cultures. Plant Cell Rep..

[18] Schmidt M.A., Tucker D.M., Cahoon E.B., Parrott W.A. (2005). Towards normalization of soybean somatic embryo maturation. Plant Cell Rep..

[19] Simmonds D.H., Donaldson P.A. (2000). Genotype screening for proliferative embryogenesis and biolistic transformation of short-season soybean genotypes. Plant Cell Rep..

[20] Tomlin E., Branch S., Chamberlain D., Gabe H., Wright M., Stewart C.N. (2002). Screening of soybean, *Glycine max* L.) Merrill, lines for somatic embryo induction and maturation capability from immature cotyledons. In Vitro Cell. Dev. Biol. Plant.

[21] Ko T.-S., Nelson R.L., Korban S.S. (2004). Screening Multiple Soybean Cultivars (MG 00 to MG VIII) for Somatic Embryogenesis Following Agrobacterium-Mediated Transformation of Immature Cotyledons. Crop Sci..

[22] Hiraga S., Minakawa H., Takahashi K., Takahashi R., Hajika M., Harada K., Ohtsubo N. (2007). Evaluation of somatic embryogenesis from immature cotyledons of Japanese soybean cultivars. Plant Biotechnol..

[23] Parrott W.A., Williams E.G., Hildebrand D.F., Collins G.B. (1989). Effect of genotype on somatic embryogenesis from immature cotyledons of soybean. Plant Cell Tissue Organ Cult..

[24] Delzer B.W., Somers D.A., Orf J.H. (1990). *Agrobacterium tumefaciens* Susceptibility and Plant Regeneration of 10 Soybean Genotypes in Maturity Groups 00 to II. Crop Sci..

[25] Bailey M.A., Boerma H.R., Parrott W.A. (1993). Genotype-specific optimization of plant regeneration from somatic embryos of soybean. Plant Sci..

[26] CSIRO (2008). Snowy—The Best Australian Soybean CSIRO.

[27] DPI (2008). Soybean-Growing Guide for Queensland—Variety Update the State of Queenzland.

[28] Gaynor L., Lawn R., James A. (2012). Agronomic studies on irrigated soybean in southern New South Wales. II. Broadening options for sowing date. Crop Pasture Sci..

[29] Watanabe S., Harada K., Abe J. (2012). Genetic and molecular bases of photoperiod responses of flowering in soybean. Breed. Sci..

[30] Shoemaker R., Amberger L., Palmer R., Oglesby L., Ranch J. (1991). Effect of 2,4-dichlorophenoxyacetic acid concentration on somatic embryogenesis and heritable variation in soybean [*Glycine max* (L) Merr.]. In Vitro Cell. Dev. Biol..

[31] Tian L.N., Brown D.C.W., Voldeng H., Webb J. (1994). In vitro response and pedigree analysis for somatic embryogenesis of long-day photoperiod adapted soybean. Plant Cell Tissue Organ Cult..

[32] Komatsuda T., Ohyama K. (1988). Genotypes of high competence for somatic embryogenesis and plant regeneration in soybean *Glycine max*. Theor. Appl. Genet..

[33] Santos K.G.B., Mundstock E., Bodanese-Zanettini M.H. (1997). Genotype-specific normalization of soybean somatic embryogenesis through the use of an ethylene inhibitor. Plant Cell Rep..

[34] Hofmann N., Nelson R., Korban S. (2004). nfluence of Media Components and pH on Somatic Embryo Induction in Three Genotypes of Soybean. Plant Cell Tissue Organ Cult..

[35] Ko T.-S., Korban S. (2004). Enhancing the frequency of somatic embryogenesis following Agrobacterium-mediated transformation of immature cotyledons of soybean [*Glycine max* (L.) Merrill.]. In Vitro Cell. Dev. Biol. Plant.

[36] Bailey M.A., Boerma H.R., Parrott W.A. (1993). Genotype effects on proliferative embryogenesis and plant regeneration of soybean. In Vitro Cell. Dev. Biol. Plant.

[37] Lazzeri P., Hildebrand D., Collins G. (1985). A procedure for plant regeneration from immature cotyledon tissue of soybean. Plant Mol. Biol. Rep..

[38] Murashige T., Skoog F. (1962). A Revised Medium for Rapid Growth and Bio Assays with Tobacco Tissue Cultures. Physiol. Plant..

[39] Gamborg O.L., Miller R.A., Ojima K. (1968). Nutrient requirements of suspension cultures of soybean root cells. Exp. Cell Res..

[40] Payne R.W. (2009). GenStat. Wiley Interdisciplinary Reviews: Computational Statistics. WIREs.

